# Developmental and Functional Brain Impairment in Offspring from Preeclampsia-Like Rats

**DOI:** 10.1007/s12035-014-9060-7

**Published:** 2015-01-10

**Authors:** Xueyuan Liu, Wenlong Zhao, Haiyan Liu, Yaoyue Kang, Chen Ye, Weirong Gu, Rong Hu, Xiaotian Li

**Affiliations:** 1 Obstetrics and Gynecology Hospital of Fudan University, 200011 Shanghai, China; 2 Shanghai Key Laboratory of Female Reproductive Endocrine Related Diseases, Shanghai, 200011 China; 3 University of Chinese Academy of Sciences, Shanghai, 200031 China; 4 Division of Infectious Disease, Huashan Hospital, Fudan University, Shanghai, China; 5 Shanghai Changzheng Hospital, Second Military Medical University, Shanghai, China; 6 Institute of Biomedical Sciences, Fudan University, Shanghai, 200011 China; 7 Shanghai Key Laboratory of Birth Defects, Shanghai, 200011 China; 8 Institute of Neuroscience, Shanghai Institutes of Biological Sciences, Chinese Academy of Sciences, Shanghai, 200031 China

**Keywords:** Preeclampsia-like, l-NAME, Water maze, Adult hippocampal neurogenesis, Gliogenesis

## Abstract

Preeclampsia is associated with developmental delay in infants and with an increased risk of various diseases in adulthood, including hypertension and epilepsy. It has been reported that several organs show developmental retardation and functional deficiency in offspring of preeclamptic rats. However, the developmental and functional changes in brains of the offspring of preeclamptic rats remain unknown. Here, we established a preeclampsia-like rat model induced using *N*ω-nitro-l-arginine methyl ester (l-NAME) to analyze the developmental and functional changes in brains of the offspring. Body and brain weights were decreased in the l-NAME group at postnatal day 0 (P0). However, there were no significant differences between the l-NAME and control groups in brain and body weights at P56. Upon further analysis, we detected a deficiency in neurogenesis, but not in apoptosis, which contributed to the smaller brains of the offspring in the l-NAME group at P0. Additionally, we observed an increase in gliogenesis to compensate for the brain weights of the offspring at P56. Although the weight and laminar structure of the brains were essentially normal at P56, spatial learning and memory were severely impaired. We also found that adult hippocampal neurogenesis was disrupted in the offspring from preeclampsia-like rats, which may explain the cognitive deficiency. Moreover, qRT-PCR revealed a reduced expression of neurogenesis-related genes in the offspring. Overall, we have described the deleterious effects of preeclampsia on the brains of offspring, providing clues to the cellular and molecular mechanisms involved in this pathogenesis, which may aid in the development of therapeutic approaches.

**Electronic supplementary material:**

The online version of this article (doi:10.1007/s12035-014-9060-7) contains supplementary material, which is available to authorized users.

## Introduction

Preeclampsia is associated with increased morbidity and mortality, and epidemiologic evidence has shown that infants from preeclamptic mothers are susceptible to respiratory distress syndrome, hypertension, stroke and/or epilepsy in adult life [1–3]. There are currently no effective strategies for preventing and treating preeclampsia, and little is known about the cellular and molecular mechanisms by which preeclampsia induces these adverse effects in offspring. Thus, it is necessary to establish preeclamptic animal models for further investigation of the adverse effects on the offspring of preeclamptic pregnancies.

Previous studies have reported that rats treated with *N*ω-nitro-l-arginine methyl ester (l-NAME), a nitric oxide synthase (NOS) inhibitor, during the last third of gestation exhibit preeclampsia-like symptoms [4, 5]. Some studies have focused on the development and function of the lungs, kidneys, and vascular endothelium of infants from preeclamptic mothers or offspring from preeclampsia animal models [6–8]. Interestingly, the mid- to late stages of pregnancy (embryonic days 14.5–20.5 in rodents; weeks 20–40 in humans), which is the timeframe in which the onset of preeclampsia occurs (approximately 20 weeks), are critical for fetal brain development in utero [9–11]. However, there are only a few scattered studies concerning the developmental and functional changes in the nervous systems of offspring from preeclampsia pregnancies in humans and rats [12, 13]. To our knowledge, the brain development and function of the offspring of preeclamptic rats have not been previously documented. Therefore, the aims of this study were to investigate whether preeclampsia disrupts the development and function of the brain and to explore the mechanisms underlying these perturbations in the offspring of preeclamptic dams.

## Materials and Methods

For detailed description of the material and methods please refer to the online-only Data Supplement.

### Animals and General Body Parameter Measurements

Adult pregnant Sprague-Dawley rats were purchased from SLAC Laboratory Animal Co. Ltd. and housed individually. All animals were given free access to food and water. Following birth, the pups were kept in the cages with their mothers. All pups were weighed with an electric scale. All procedures were approved by the Animal Care and Use Committee of the University of Fudan.

### Establishment of the Preeclampsia-Like Rat Model

The pregnant dams were randomly divided into two groups, which were given 50 mg kg^−1^ day^−1^ of l-NAME or pure water via daily gavage from day 14.5 to day 20.5 of gestation [6]. Their systolic blood pressure (SBP) and urokinase protein levels were detected with a BP-2000 Blood Pressure analyzer (BP-2000, Visitech Systems Inc., North Carolina, USA) and a Bayer ADVIA 1650 analyzer (ADVIA 1650, Bayer, Leverkusen, Germany), respectively.

### Water Maze Test

We used 15 P56 male rats from the l-NAME treatment group and 15 P56 rats from the control group in the water maze test. And it was carried out as described previously [14] but with modification as described in the Data Supplement.

### Immunohistochemistry

The P0 rat brains were sliced into 20-μm coronal sections on glass slides, while the P56 rat brains were sliced to 30-μm free-floating coronal sections. And images were acquired with a SP8 confocal microscope (Leica Microsystems, Wetzlar, Germany). The information about the primary and secondary antibodies and the applied working concentrations are provided in Supplemental Table 1.

### Hematoxylin and Eosin Staining

The slices were treated with a hematoxylin and eosin staining kit (Beyotime, C0105, Wuhan, China) following the manufacturer’s instructions.

### Quantitative Real-Time PCR Assays

Total cortical RNA from P0 rats or the hippocampus of P56 rats was subjected to extraction using TRIzol (Invitrogen, 15596026, Carlsbad, CA, USA). Reverse transcription and first-strand cDNA synthesis were performed using the PrimeScript™ RT reagent kit (TaKaRa, RR047A, Japan). Quantitative real-time PCR was carried out with the EvaGreen dye (Biotium, catalog # 31000, USA). The primers used in the qRT-PCR assays were specific for neurogenesis-associated genes. The primer sequences are listed in Supplemental Table 2.

### Statistical Analyses

Statistical calculations were conducted using GraphPad Prism 5 software. In the statistical graphs, the error bars represent the s.e.m. Statistical significance was determined using Student’s *t* test, the Mann-Whitney test, or one-way ANOVA, and *P* values < 0.05 were considered statistically significant.

## Results

### Establishment of the Preeclampsia-Like Rat Model

**Fig. 1:**
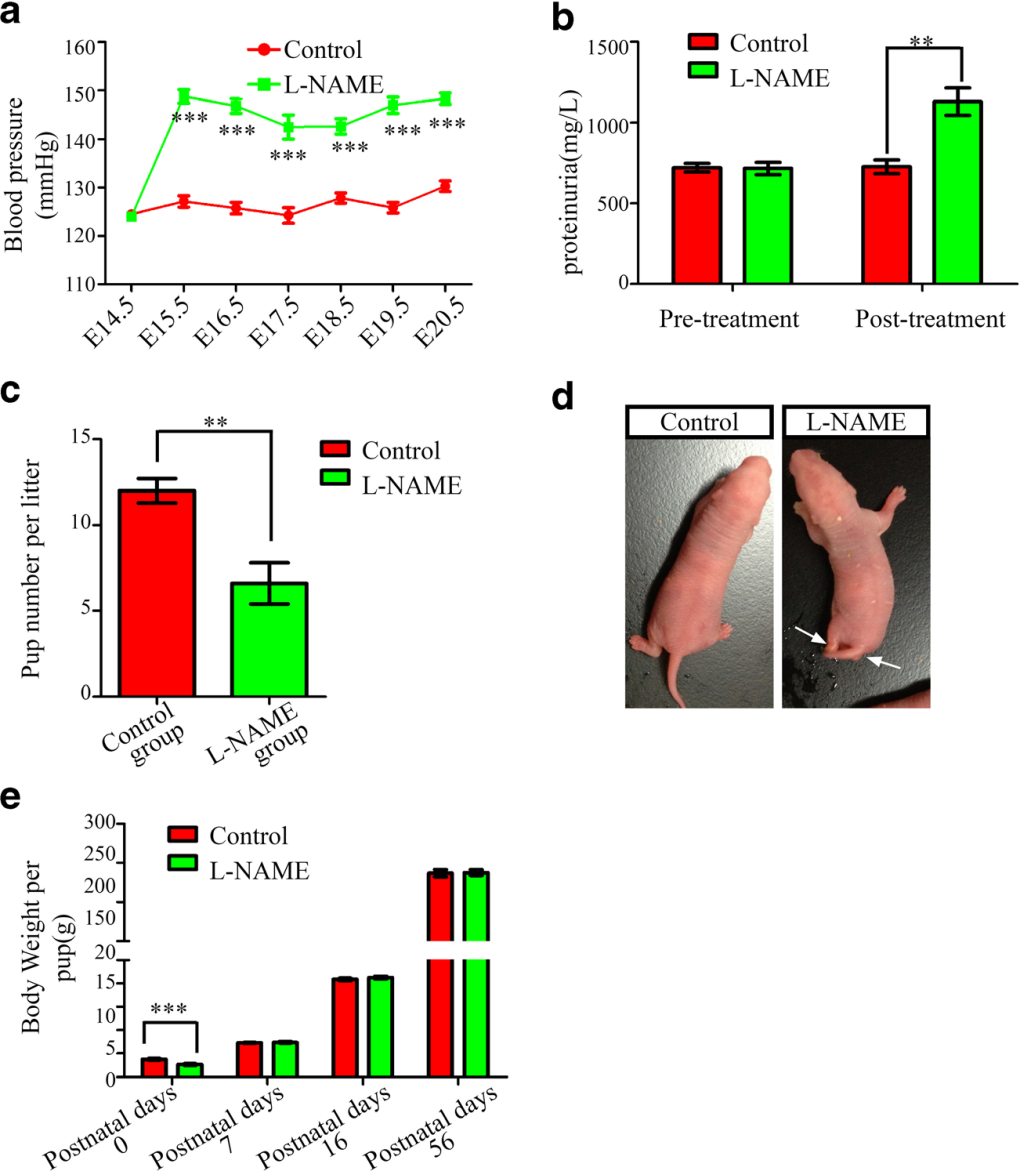
Establishment of a preeclampsia-like model in rats. **a** Systolic blood pressure of pregnant rats in the l-NAME and control groups. The mean ± s.e.m. is shown. Statistical analysis was performed using Student’s *t* test. **b** Urokinase protein levels in pregnant rats in the l-NAME and control groups. The mean ± s.e.m is shown. Statistical analysis was performed with the Mann-Whitney test. **c** Number of rat pups per litter in the l-NAME and control groups. The mean ± s.e.m. is shown. Statistical analysis was performed with the Mann-Whitney test. **d** Characteristic gross anatomic pathologic findings in P0 rats from preeclampsia-like mothers compared with control mothers. *Arrows* indicate limb defects. **e** Body weight at P0, 7, 16, and 56 of rat pups in the l-NAME and control groups. The mean ± s.e.m. is shown. Statistical analysis was performed with the Mann-Whitney test. **P < 0.01; ***P < 0.001

To detect the consequences of preeclampsia in the brains of rat offspring, we first examined the perinatal effects of treating pregnant rats with l-NAME to confirm whether we had successfully established a preeclampsia-like animal model. We found that l-NAME administration resulted in increased blood pressure levels associated with proteinuria in the pregnant rats (Fig. 1a, b). Then, we obtained 84 newborn rat pups from the control group and 56 from the l-NAME group. We found that in the l-NAME group, the fetal mortality rate was increased (Fig. 1c), and two pups exhibited hindlimb necrosis (Fig. 1d). Excluding the dead pups and those with hindlimb necrosis from further analyses, the body weight at birth was significantly lower in the l-NAME group [5.332 ± 1.186 g] than in the control group [7.503 ± 1.341 g]; *p* < 0.001 (Fig. 1e). However, there were no significant differences in body weight between the two groups from postnatal day 7 onward (Fig. 1e). These results were consistent with previous reports [6] and suggested that our preeclampsia-like animals were suitable for further analysis of the developmental and functional changes in the brains of offspring.

### Evaluation of Brain Development in the Offspring of Pregnant Rats Treated with l-NAME

**Fig. 2:**
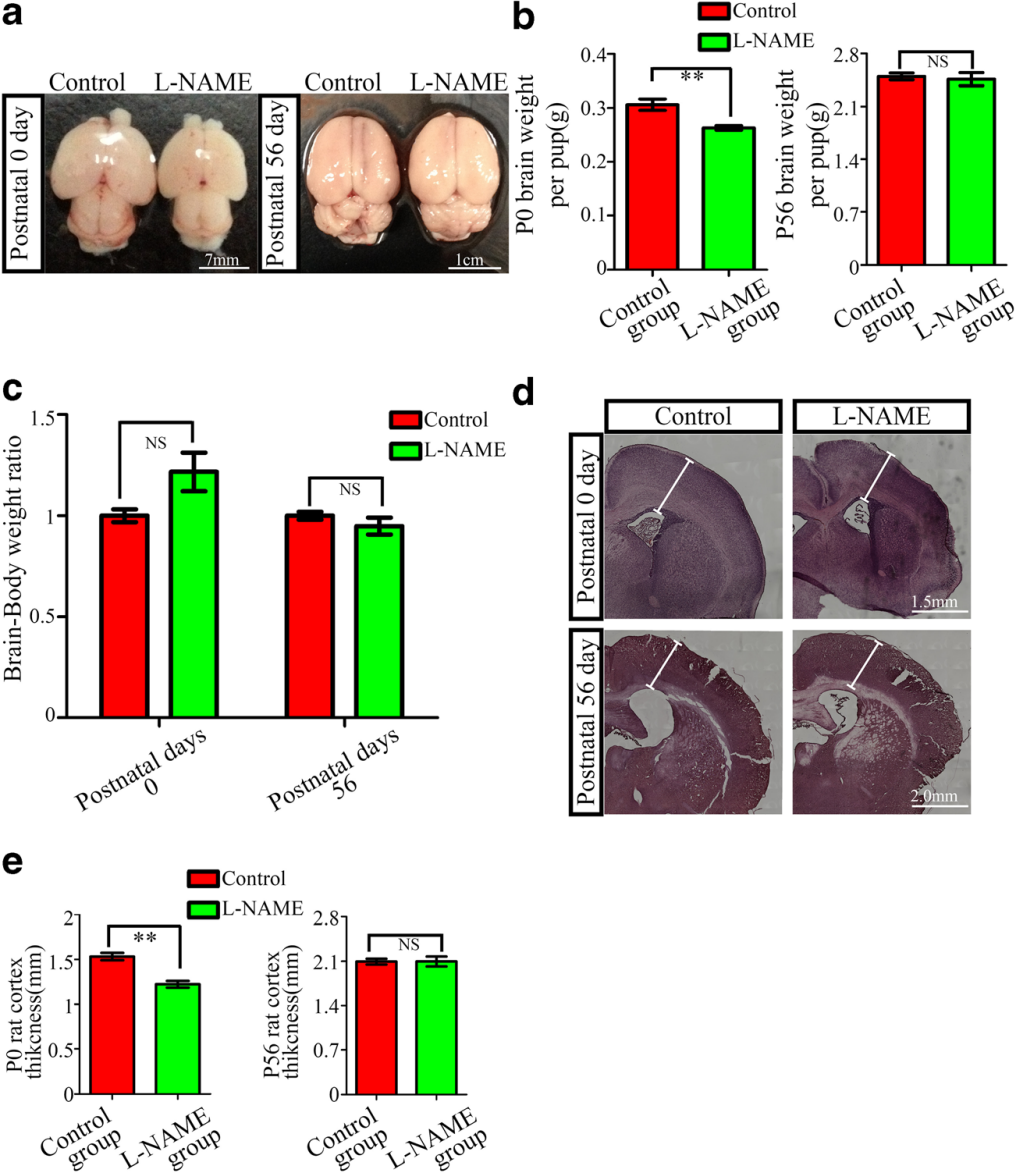
Evaluation of brain development in offspring from the l-NAME group. **a** Dorsal view of P0 (*left panel*) and P56 (*right panel*) rat brains in the l-NAME and control groups. **b** Brain weight at P0 and P56 in the l-NAME and control groups. The mean ± s.e.m is shown. Statistical analysis was performed with the Mann-Whitney test. **c** Ratio of brain weight to body weight at P0 and P56 in the l-NAME and control groups. **d** Coronal sections of P0 (*upper panel*) and P56 (*lower panel*) rat brains stained with hematoxylin and eosin. **e** Statistical results for cortex thickness in P0 and P56 rat pups in the l-NAME and control groups. The mean ± s.e.m. is shown. Statistical analysis was performed using Student’s *t* test. **P < 0.01

We found that the offspring in the l-NAME group exhibited smaller brains at P0, but they were not significantly different at P56 compared with the control group (Fig. 2a, b). Furthermore, the ratio of the brain weight to body weight was unchanged in the l-NAME group (Fig. 2c). Hematoxylin and eosin staining showed that the radial dimension (thickness) was decreased at P0, but not at P56, in the l-NAME group compared with the controls (Fig. 2d, e). These results indicated that the growth of the whole body, including the brain, is delayed prenatally, but this developmental retardation could recover after delivery.

**Fig. 3:**
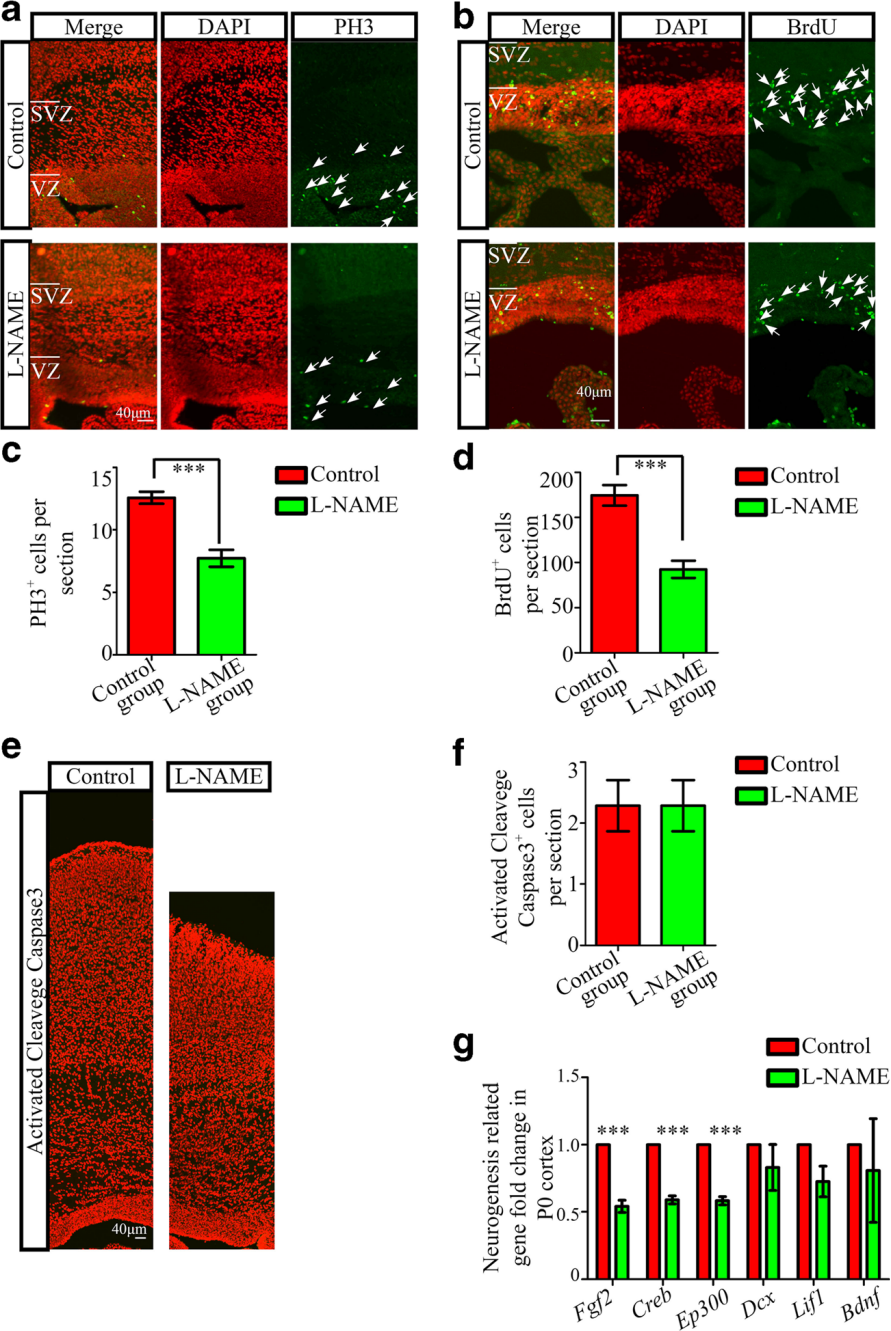
Analysis of progenitor cell proliferation and apoptosis in newborn offspring in the l-NAME and control groups. **a** PH3 immunofluorescence in coronal sections of the neocortex. **b** BrdU immunofluorescence in coronal sections of the neocortex after 4 h of BrdU incorporation. **c** Number of PH3-positive cells in the VZ per section. **d** Number of BrdU-positive cells in the VZ per section. **e** Immunofluorescence staining of coronal sections of the neocortex with an antibody against activated cleavage caspase3. **f** Number of activated cleavage caspase3-positive cells per section. **g** mRNA levels of neurogenesis-related genes at P0 rat offspring from the l-NAME and control groups, as detected via qRT-PCR. *VZ* ventricular zone, *SVZ* subventricular zone. *N* > 3/3, the number of sections examined from at least three rats each group. The mean ± s.e.m. is shown. Statistical analysis was performed using Student’s *t* test. ***P < 0.001

To elucidate the cellular and molecular mechanisms underlying the defects in early brain development observed in the l-NAME group offspring, we evaluated proliferation and apoptosis in P0 rats through immunostaining for PH3 and activated cleavage caspase3, respectively. Acute BrdU incorporation assays were also performed to examine cell proliferation. We evaluated the numbers of PH3^+^ cells as well as BrdU^+^ and activated cleavage caspase3^+^ cells at P0 within the ventricular zone (VZ) of the neocortex. Interestingly, we observed a decrease in the numbers of PH3^+^ and BrdU^+^ cells in the neocortex in the l-NAME group (Fig. 3a–d). However, the number of activated cleavage caspase3^+^ cells was not significantly different between the two groups (Fig. 3e, f). To investigate the mechanism at the molecular level, we examined the expression levels of several genes involved in the proliferation of radial glia, including *Fgf2* and others listed in Supplemental Table 2 [15]. Using RT-PCR, we detected reduced expression of the *Fgf2*, *Creb*, and *Ep300* genes in the l-NAME group compared with the control group (Fig. 3g).

**Fig. 4:**
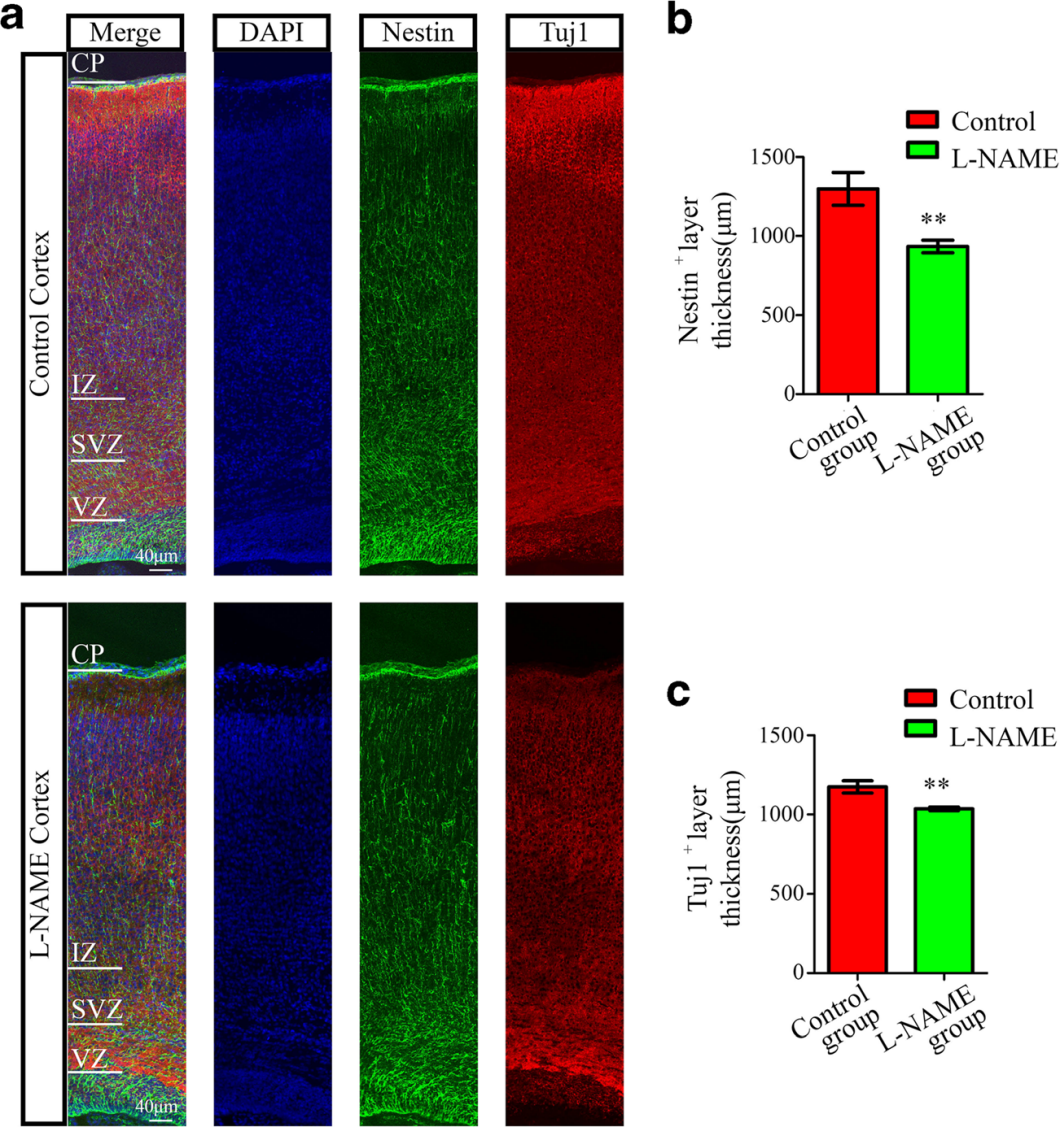
The radial glial scaffold of newborn offspring in the l-NAME and control groups. **a** Immunofluorescence images of Nestin and Tuj1 in coronal sections of the neocortex. **b** Thickness of the Nestin-positive region at P0 rat offspring from the l-NAME and control groups. **c** Thickness of the Tuj1-positive region at P0 rat offspring from the l-NAME and control groups. *VZ* ventricular zone, *SVZ* subventricular zone, *IZ* intermediate zone, *CP* cortical plate. *N* > 3/3, the number of sections examined from at least three rats each group. The mean ± s.e.m. is shown. Statistical analysis was performed using Student’s *t* test. **P < 0.01

We next questioned whether l-NAME treatment affected the number and structure of radial glia, which are recognized as neural stem cells and provide the scaffold for the migration of newborn neurons. We analyzed the morphological changes in the radial glial scaffold and the differentiation of neural progenitor cells via immunostaining for the cell-type-specific markers Nestin and Tuj1, respectively. We found that the morphology of the radial glia scaffold in the l-NAME group was similar to that in the control group. However, we discovered that the Nestin^+^ regions and Tuj1^+^ regions in the l-NAME group were thinner than in the control group (Fig. 4a–c). Therefore, we concluded that the lower brain weights and smaller brain sizes may have resulted from a deficiency in the proliferation of neural progenitor cells in early developmental stages, without affecting the differentiation of neural progenitor cells or the morphology of the radial glial scaffold.

Next, we investigated the cellular mechanism underlying the recovery observed in adult brains in the l-NAME group. First, we examined the laminar structure of adult brains via immunostaining for Ctip2 and Tbr1, which are deeper layer and thinner layer markers, respectively. We found that the patterns of the Ctip2^+^ and Tbr1^+^ cell layers were similar between the two groups (Fig. S1a, b).

**Fig. 5:**
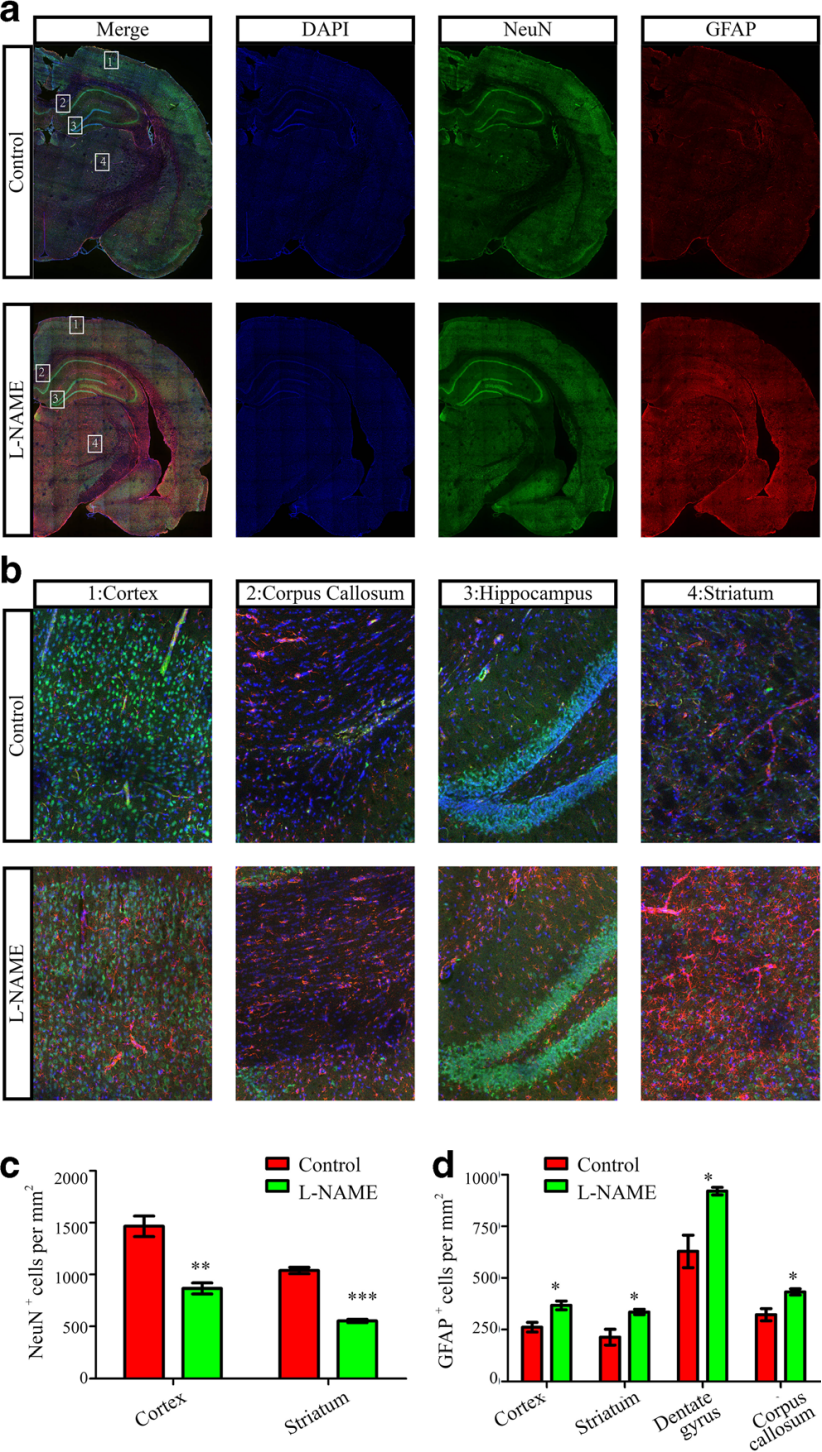
Quantification of neurons and astrocytes in P56 offspring from the l-NAME and control groups. **a** NeuN and GFAP immunofluorescence in coronal sections of the neocortex from the l-NAME and control groups. **b** Enlarged images from regions 1, 2, 3, and 4 in **a**. Region 1, cortex; region 2, corpus callosum; region 3, hippocampal DG; region 4, striatum. **c** The number of NeuN-positive cells in the neocortex and striatum per section in rat offspring from the l-NAME and control groups. **d** The number of GFAP-positive cells in the neocortex, striatum, dentate gyrus, and corpus callosum per section in rat offspring from the l-NAME and control groups. *N* > 3/3, the number of sections from at least three rats of each group. The mean ± s.e.m. is shown. Statistical analysis was performed using Student’s *t* test. *P < 0.05, **P < 0.01, ***P < 0.001

Glial cells constitute nearly 50 % of the cells in the mammalian brain, and astrocytes, which proliferate postnatally, are the largest glial population [16]. Thus, we explored the numbers of neurons and astrocytes in adult brains through immunostaining for the neuronal marker NeuN and the astrocytic marker GFAP. Intriguingly, we found that the number of GFAP^+^ cells was increased in the whole adult brains from the l-NAME group following reduction of the number of NeuN^+^ cells (Fig. 5a–d). These data suggested that the proliferation of astrocytes may be a contributing factor in the recovery of brain weight among the offspring of preeclamptic dams.

### Impairment of Learning and Memory in the Offspring of the l-NAME Group

**Fig. 6:**
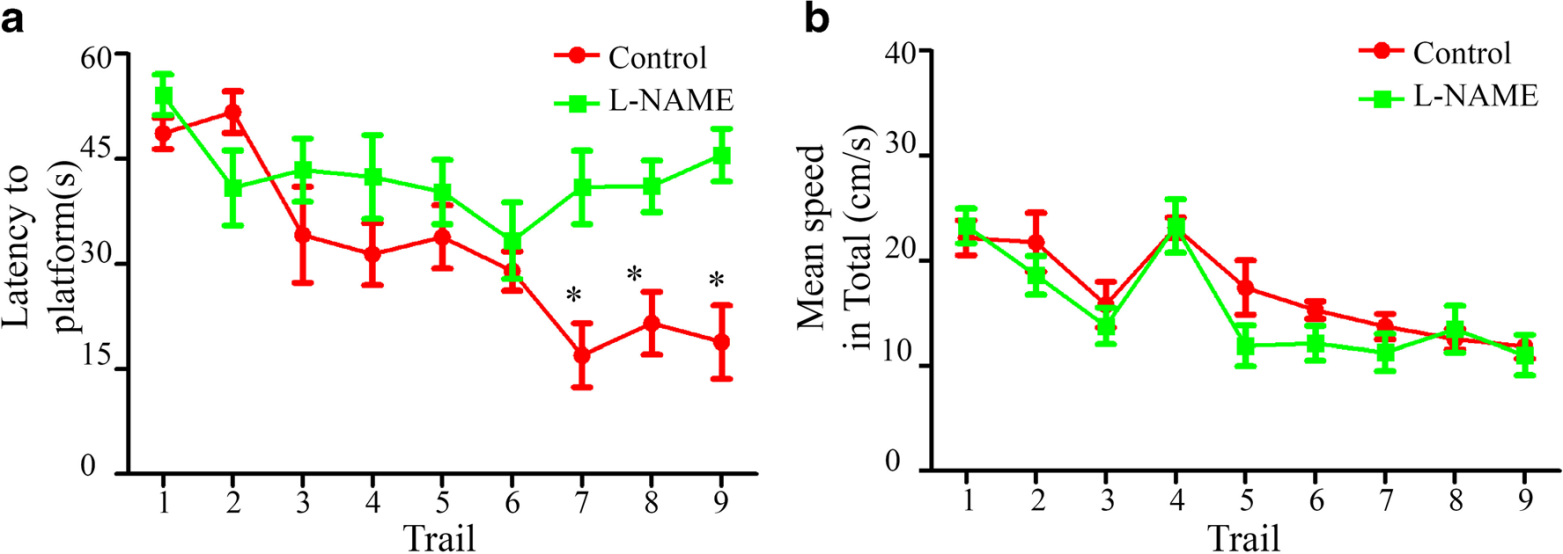
Spatial learning is impaired in offspring from preeclamptic pregnancies. **a** Latency to escape onto the hidden platform in rat offspring from the l-NAME and control groups during training trials. **b** Mean speed during the total training time (1 min) in rat offspring from the l-NAME and control groups. The mean ± s.e.m. is shown. Statistical analysis was performed using Student’s *t* test. *P < 0.05. *N* = 15/15, number of rats tested in training trials from the control and l-NAME groups, respectively

Although the brain weight and laminar structure were essentially normal in the offspring of the preeclampsia-like rats at adulthood, we investigated the existence of possible functional impairment. We selected P56 male offspring from the control and l-NAME groups to analyze their learning and memory abilities using the water maze test. During the training stage, the results revealed that the rat offspring from the control group progressively learned the task from session 1 to session 9, whereas the rat offspring from the l-NAME group were markedly slower and less efficient at learning the task (Fig. 6a). However, no significant differences in swimming speed were found between the two groups (Fig. 6b). Thus, the spatial learning abilities of rat offspring from preeclamptic pregnancies are impaired.

**Fig. 7:**
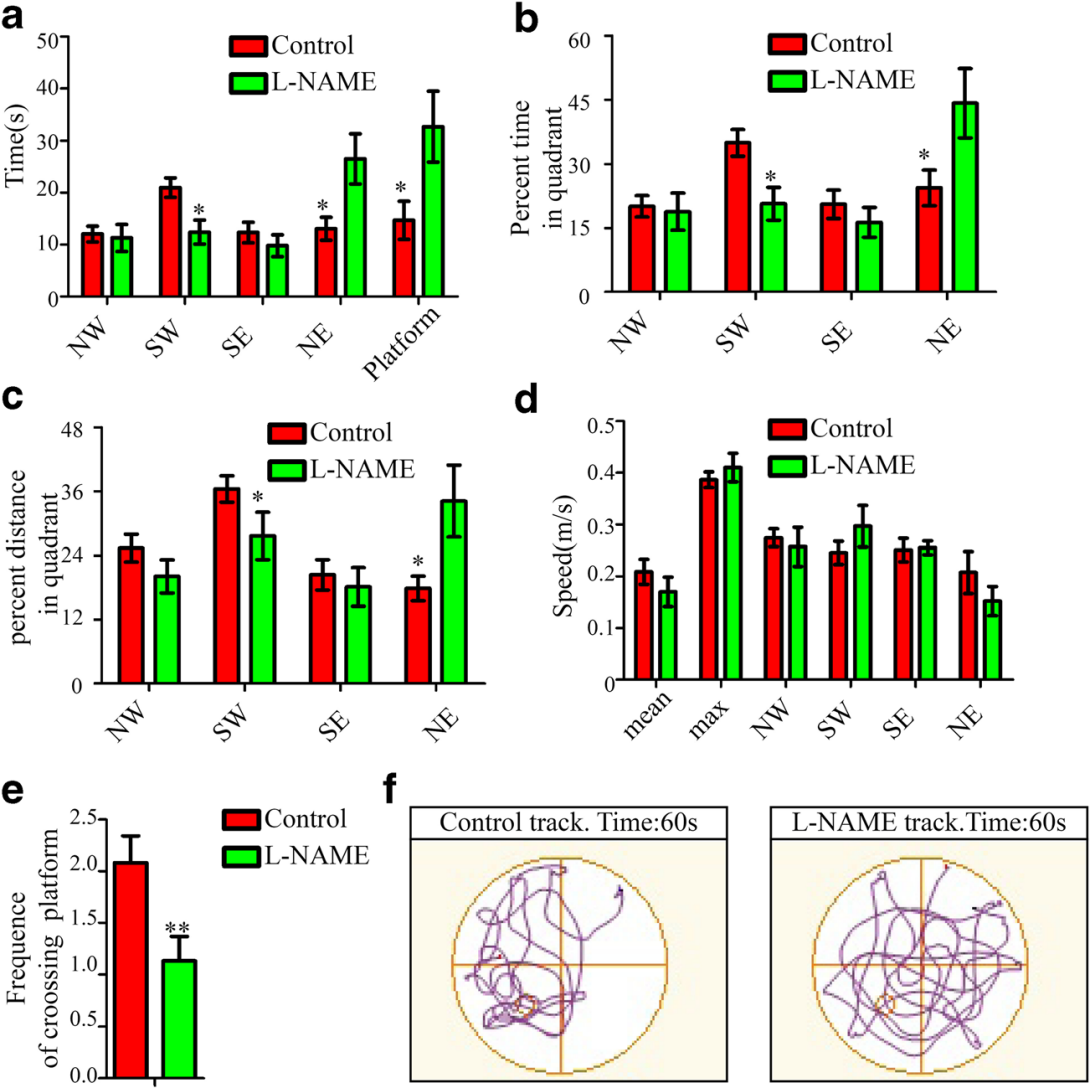
Spatial memory is impaired in offspring from preeclamptic pregnancies. **a** Time spent in each quadrant and latency to escape onto the training platform area of the Morris water maze in the probe trial in the l-NAME and control groups. **b** Percentage of time spent in each quadrant of the Morris water maze during the probe trial in the l-NAME and control groups. **c** Percentage of the distance swum in each quadrant of the Morris water maze during the probe trial in the l-NAME and control groups. **d** Mean and maximum speeds in the total probe time as well as the mean speed in each quadrant in the l-NAME and control groups. **e** Frequency of crossing over the platform area in the l-NAME and control groups in probe trial. **f** Typical behavior tracks of the tested rat offspring in the l-NAME and control groups. The mean ± s.e.m. is shown. Statistical analysis was performed using Student’s *t* test. *P < 0.05; ** *P* < 0.01. *N* = 15/15, numbers of rats in the test trials from the control and l-NAME groups, respectively

At the probe stage, the results revealed that the rat offspring from the control group spent more time in the target quadrant and less time in the target contra-quadrant than the offspring from the l-NAME group (Fig. 7a). And offspring from the l-NAME group displayed significantly higher latencies to escape to the training platform area compared with the control group (Fig. 7a). Additionally, we found that the frequency of platform crossing in the control group was higher than that in the l-NAME group (Fig. 7e, f). Higher percentages of time and a greater swimming distance within the target quadrant were observed in the control group than in the l-NAME group (Fig. 7b, c). The recorded swimming speeds, including the mean speed, maximum speed, and the speeds in the four quadrants, did not show differences between the two groups (Fig. 7d). Therefore, we have demonstrated that the rat offspring from preeclamptic pregnancies display deficiencies in spatial learning and memory.

### Impairment of Adult Hippocampal Neurogenesis in Offspring from the l-NAME Group

**Fig. 8:**
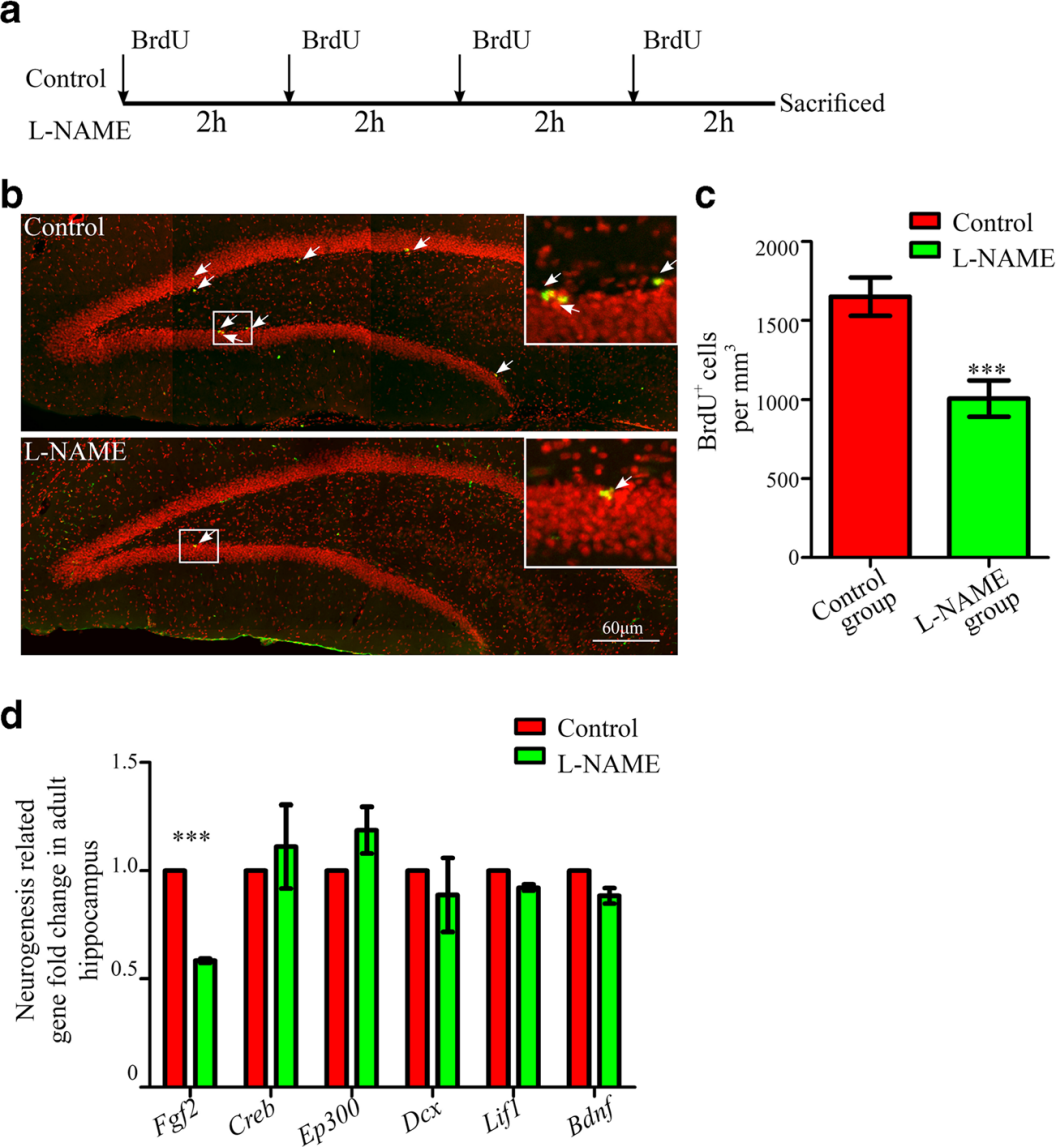
Adult hippocampal neurogenesis is impaired in offspring from preeclamptic pregnancies. **a** Scheme of BrdU solution administration in offspring in the l-NAME and control groups. **b** Typical cases showing BrdU-labeled cells in the adult hippocampus in the l-NAME and control groups following immunofluorescence staining with a BrdU antibody (*green*) and staining with DAPI (*red*). *Arrows* indicate BrdU-positive cells. **c** Total BrdU per mm^3^ in the adult hippocampus in the offspring of l-NAME and control rats. **d** Expression levels of neurogenesis-related genes in the adult offspring of l-NAME and control rats, as detected via qRT-PCR. The mean ± s.e.m. is shown. Statistical analysis was performed with Student’s *t* test. **P < 0.01, ***P < 0.001. NS, *P* > 0.05. *N* = 3/3, numbers of rats examined in these assays from each group

To reveal the cellular and molecular mechanisms underlying the functional impairment of brain function in the offspring of preeclamptic rats, we examined adult hippocampal neurogenesis, which is associated with the disruption of spatial learning and memory abilities [17]. Thus, the rat offspring were injected with BrdU solution, as shown in a schematic diagram (Fig. 8a). We found that the numbers of BrdU^+^ cells in the adult hippocampus were dramatically decreased in the l-NAME group (Fig. 8b, c). Next, we used RT-PCR to detect the expression levels of genes associated with adult hippocampal neurogenesis [15]. We observed under-expression of the *Fgf2* gene in the l-NAME group (Fig. 8d). This defect in adult hippocampal neurogenesis may be the cellular and molecular mechanism underlying the deficiency in spatial learning and memory in offspring from the l-NAME group.

## Discussion

In the present study, we confirmed that treatment of rats with the NOS inhibitor l-NAME from E14.5 to birth, which is a critical stage for brain development, could cause a preeclampsia-like syndrome and IUGR in the offspring. At birth, body weight was lower in the l-NAME group than in the control group, and the l-NAME group showed a significant delay in brain development. l-NAME administration may have interfered with brain development simply by impairing maternofetal exchange, thereby reducing the nutrition available to the offspring, since the brain is a fetal organ with the highest metabolism, consuming the largest amount of nutrients during development. And in previous studies, poor nutrition has been shown to result in poor neurodevelopment by impairing neurogenesis or other cellular processes [18]. In our rat model, we observed a disruption of neurogenesis in the offspring from the l-NAME group. Moreover, growth factors are crucial for translating nutritional fuel into brain growth through a series of signaling pathway cascades [19]. The production of growth factors can be inhibited by a lack of nutrients [20]. Interestingly, in offspring from the l-NAME group at P0, we detected a reduced expression of *Creb*, *Ep300*, and *Fgf2*, which are important for brain growth and neurogenesis. Therefore, increasing nutrient supplementation or providing some specific critical nutrition component may be beneficial for the in utero brain development of offspring of preeclamptic mothers.

Interestingly, we found that the low body weight and brain weight were recovered approximately 1 week after birth. The results concerning body weight were consistent with previous data. The observed rapid growth that abolished the differences in brain size were similar to the findings described in a previous report addressing lung development in rats showing decreases in lung volume at P0 that were resolved within the first 2 postnatal weeks [6]. These results are also consistent with observations from human cases. Babies from preeclamptic pregnancies show a reduced head circumference and a low body weight at age 2 years [21]. There are some reports showing rapid recovery in body weight or head circumference in children or adult humans, which may be due to sufficient nutrient uptake after birth. Thus, we propose that the fetus might receive cues from the mother’s preeclamptic state and transiently alter its metabolism, hormone production, tissue sensitivity to circulating factors, and even the expression of certain genes to adapt to the mother’s health while in utero. After delivery, the development of pups is partially restored to normal without these cues from mothers, which might be the reason for the rapid recovery observed in the body weight and development of organs.

It is unfortunate that the unique developmental pattern of the brain may not completely recover. First, embryonic brain development is critical for the neurodevelopment of vertebrates throughout life. In rodents, several pivotal neurodevelopment processes occur between E14 and E20 parallel to the onset of preeclampsia in humans. And the developmental pattern is similar in humans. These processes include embryonic neurogenesis, neuronal migration, neuronal morphogenesis, and gliogenesis which produces minority of glia and/or glial precursors [9, 22]. Postnatally, the major neurodevelopment processes in both rodents and humans are gliogenesis and experience-dependent synapse remodeling/pruning [23, 10]. Although the offspring have separated from the mother at this stage and may obtain appropriate nutrition, certain biological events such as neurogenesis, which predominantly occurs during the embryonic stage, cannot be repaired at postnatal stage. In fact, we observed overproduction of astrocytes, which may contribute to the recovery of brain weight and size, and the numbers of neurons in two brain regions in the offspring of preeclampsia-like rats remained lower than in normal brains.

In addition, extensive studies have shown that the functions of many organs or tissues, such as vascular and renal endothelia, are impaired or dysfunctional in the offspring of mothers with preeclampsia or other diseases, showing no resolution through sufficient nutrition or other supplementation after birth [24, 8, 25]. In the present study, the rats from the preeclampsia-like group exhibited defects in spatial learning and memory, although no macroscopic defects were observed in the brains of the adult offspring from this group. In humans, one paper has reported that the hypertensive diseases of pregnancy, including gestational hypertension and preeclampsia, are associated with neurocognitive outcomes in middle childhood. The authors of this work found that scores on the Peabody Picture Vocabulary Test-Revised (PPVT-R) are lower in offspring from pregnancies with maternal hypertension than in children from normotensive pregnancies [12].

Furthermore, we observed a deficiency in neurogenesis in the adult hippocampus, which may lead to the defective spatial learning and memory detected in the l-NAME group. Over the last decade, many studies have shown that new neurons can be continually generated in the subventricular zone of the lateral ventricles (SVZ) and in the subgranular zone of the hippocampal dentate gyrus (SGZ). The newborn neurons from the SVZ migrate into the olfactory bulb, which contributes to olfactory sensation in mammals [26]. The newborn neurons in the SGZ of the dentate gyrus incorporate into the molecular layer and integrate into neuronal circuits involved in learning and memory and in other cognitive processes [27, 28]. The deficiency in adult hippocampal neurogenesis may cause the defects in spatial learning and memory observed in rat offspring from preeclampsia-like pregnancies. Alternatively, astrocytes are crucial to the regulation of synaptic connectivity and function. Therefore, we do not rule out a contribution of the rapid and robust generation of astrocytes in the DG region to the defective spatial learning and memory detected in the water maze test.

However, the mechanisms underlying these long-term effects in offspring following l-NAME-induced preeclampsia remain unknown. We propose two possible explanations for these consequences.

First, accumulated evidence has indicated that adult neural stem cells, including adult hippocampal neural stem cells, are derived from radial glia in the embryo [29, 30]. Additionally, we observed that the offspring from the l-NAME group rats show deficiencies in cell proliferation in the VZ at P0 and in neurogenesis in the adult hippocampus. Thus, we speculate that the number of embryonic radial glia, which would develop into adult stem cells in the hippocampus, is reduced after l-NAME administration.

Second, genes participating in the proliferation of adult stem cells are downregulated in offspring from preeclampsia-like rat. We observed that the mRNA levels of genes involved in proliferation, such as *Fgf2*, *Creb*, and *Ep300*, were decreased in P0 cortex and/or adult hippocampus in the offspring from the l-NAME group [31, 32]. *Fgf2* belongs to the family of fibroblast growth factors, which are known to play critical roles in cell proliferation and differentiation. *Fgf2* ^−/−^ mice have been shown to present an approximately 10 % reduction in cerebral cortex size and neuronal density, and these mice show impairments in memory at adulthood [33–35]. Furthermore, these mice exhibited defects in spatial learning and memory. Interestingly, it has been reported that *Fgf2*, *Creb*, and *Ep300* are involved in pathogenesis of epilepsy which is susceptive in offspring from preeclamptic mothers [36–38]. And localized overexpression of *Fgf2* could reduce epileptogenesis in an epilepsy rat model. Therefore, the changes in expression levels of the above genes may contribute to the deficiencies in adult hippocampal neurogenesis and in cell proliferation observed in P0 rats following the administration of l-NAME.

Moreover, the expression levels of *Fgf2*, *Creb*, and other genes are known to be dynamically regulated during development and in response to environmental cues. It has been reported that the expression of *Fgf2* can be regulated by modifying the methylation status within gene promoters [39, 40]. However, we did not detect any difference in methylation status within the *Fgf2*, *Creb*, and *Ep300* promoters between the control and l-NAME groups (data not shown). Further analysis is necessary to reveal the underlying mechanism in future works.

Taken together, we found perturbations in neurogenesis and in spatial learning and memory as well as gliosis in offspring from preeclampsia-like rats. The deficiencies in adult hippocampal neurogenesis, with under-expression of related genes, and gliosis provide insight into the mechanisms causing the deficiencies in spatial learning and memory. The combined results of our study may provide a plausible model for elucidating the influence and mechanisms of maternal preeclampsia on human infants and suggest alerting the status of neurogenesis might be a promising approach to improve the long-term prognosis of offspring from preeclamptic pregnancy.

## Electronic Supplementary Material

Below is the link to the electronic supplementary material.

Supplemental Fig. 1 Analysis of the neocortical layers of P56 offspring in the l-NAME. (A) Immunofluorescence images of Ctip2 in coronal sections of the neocortex in l-NAME group. The middle and right panels are enlarged images from regions 1 and 2 in the left panel (B) Immunofluorescence staining of coronal sections of the neocortex with a Tbr1 antibody in l-NAME group. The middle and right panels are enlarged images from regions 1 and 2 in the left panel. (JPEG 5406 kb)

ESM 2 (PDF 194 kb)

ESM 3 (XLS 16 kb)

ESM 4 (XLS 24 kb)
