# Supplementary figures and images for: Developmental and Functional Brain Impairment in Offspring from Preeclampsia-Like Rats
Source: Mol Neurobiol. 2015 Jan 10;53(2):1009–19. doi: 10.1007/s12035-014-9060-7 (PMC4752589; doi:10.1007/s12035-014-9060-7)

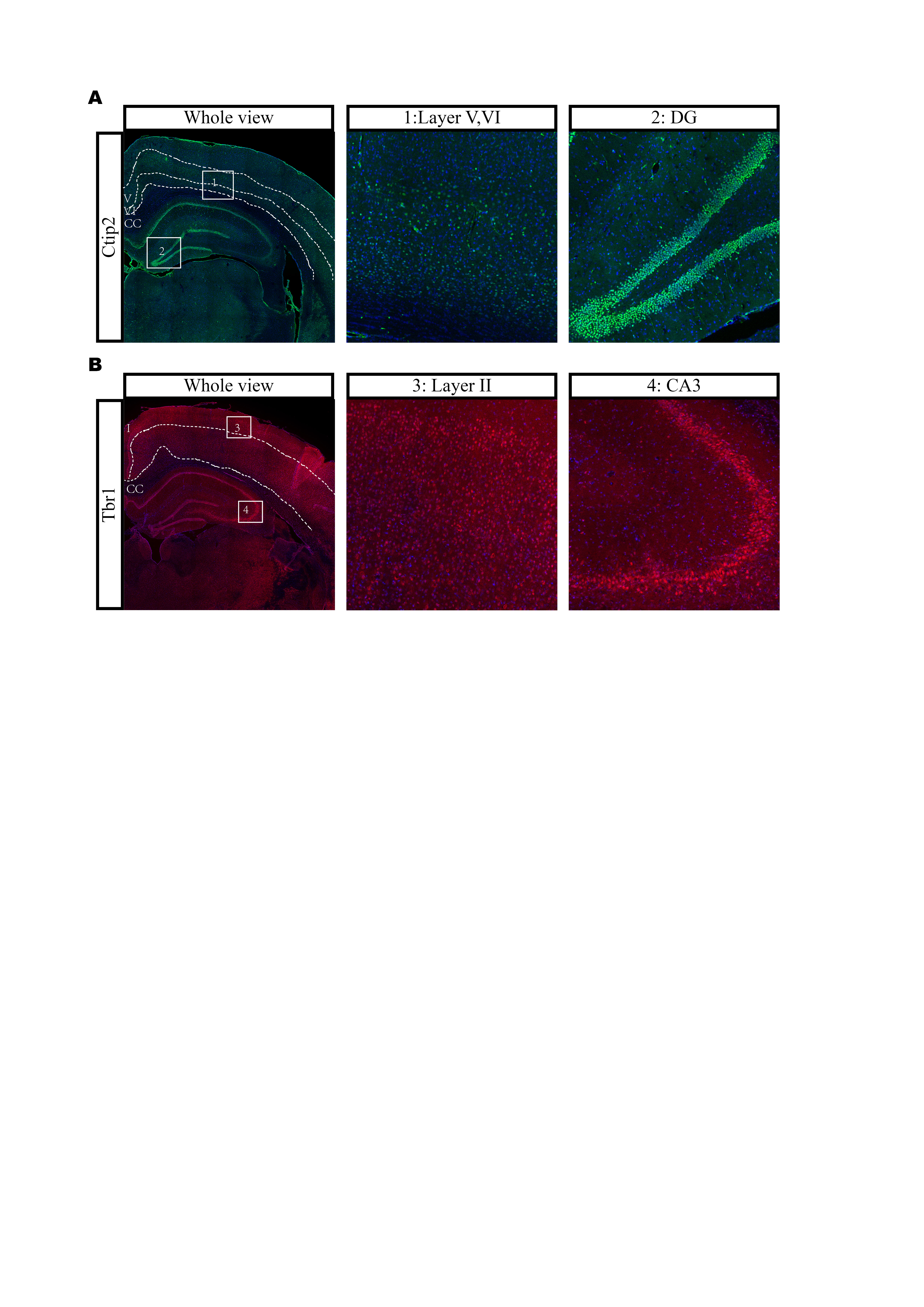

Supplement: Supplementary file 1 — Analysis of the neocortical layers of P56 offspring in the l-NAME. (A) Immunofluorescence images of Ctip2 in coronal sections of the neocortex in l-NAME group. The middle and right panels are enlarged images from regions 1 and 2 in the left panel (B) Immunofluorescence staining of coronal sections of the neocortex with a Tbr1 antibody in l-NAME group. The middle and right panels are enlarged images from regions 1 and 2 in the left panel. (JPEG 5406 kb) [file 12035_2014_9060_Fig9_ESM.jpg]
