# Supplementary material for: Developmental and Functional Brain Impairment in Offspring from Preeclampsia-Like Rats
Source: Mol Neurobiol. 2015 Jan 10;53(2):1009–19. doi: 10.1007/s12035-014-9060-7 (PMC4752589; doi:10.1007/s12035-014-9060-7)
Supplement: Supplementary file 2 — (PDF 194 kb) [file 12035_2014_9060_MOESM1_ESM.pdf]

Materials and methods on-line:

Animals and general body parameter measurements

Adult pregnant Sprague-Dawley rats were purchased from SLAC Laboratory Animal Co. Ltd. and housed individually. All animals were given free access to food and water. Following birth, the pups were kept in the cages with their mothers. All pups were weighed at birth and on postnatal days 7, 16 and 56 with an electric scale. Some pups were sacrificed at birth or on postnatal day 56 for brain weight measurement and further analysis.

All procedures were approved by the Animal Care and Use Committee of the University of Fudan.

Establishment of the preeclampsia-like rat model

The pregnant dams were randomly divided into two groups, which were given 50 $mg \cdot kg^{-1} \cdot d^{-1}$ L-NAME or pure water via daily gavage from day 14.5 to day 20.5 of gestation[6]. Their systolic blood pressure (SBP) and urokinase protein levels were detected with a BP-2000 Blood Pressure analyzer (BP-2000, Visitech Systems Inc., North Carolina, USA) and a Bayer ADVIA 1650 analyzer (ADVIA 1650, Bayer, Leverkusen, Germany), respectively.

Water maze test

For the water maze test, we used 15 P56 male rats from the L-NAME treatment group and 15 P56 rats from the control group. For the spatial learning stage, a circular platform (8 cm in diameter) was placed at a specific location away from the edge of the pool. The top of the platform was submerged 1.5 cm below the water surface. The animals were trained in three trials per day. In one trial, the animals were placed at a certain position and given 60 s to find the platform. If the animal could not find the platform, it was guided to the platform and was allowed to stay on the platform for 20 s. The training procedure lasted for 4.5 days, and 9 trials were conducted in total.

For the memory retention test, the above trained animals were placed at a specific position in the pool with the platform removed and given 60 s to swim. The time they spent in each quadrant (target quadrant, left quadrant, opposite quadrant and right quadrant), the first latency to the platform area and the frequency of reaching the platform area were recorded. All data from the water maze test were collected with a video camera fixed to the ceiling and connected to a computer and a video-tracking system (Noldus Information Technoloy, Holland), both located in an adjacent room.

Immunohistochemistry

P0 and P56 rats were anesthetized with chloral hydrate and perfused with 4% paraformaldehyde, and their brains were post-fixed overnight at 4℃. The P0 rat brains were sliced into 20 μm coronal sections on glass slides, while the P56 rat

brains were sliced to 30 μm free-floating coronal sections. The slices were blocked in blocking buffer (10% BSA, 0.2% Triton X-100 in PBS) for 1 h at room temperature. To perform BrdU staining, the sections were submerged in 96-100℃ antigen retrieval buffer (11.6 mMol trisodium citrate dehydrate, 2 mMol citric acid, pH=6.8) for 10 min. After slowly cooling the antigen retrieval buffer to room temperature, the sections were treated with 2 N HCl at 37 ℃ for 12 min for P0 brain slices or 20 min for P56 brain slices, followed by washing with $Na_2Ba_4O_7$ (0.1 M, pH 8.5) for 10 min and washing with PBS three times for 10 min each. Then, the slices were incubated with primary antibodies overnight at 4℃. Information about the primary antibodies and the applied working concentrations are provided in Supplemental Table 1. After incubation with the primary antibodies and three 10 min washes in PBS, the slices were incubated with fluorescently conjugated IgG Alexa 488 and/or Alexa 546 at room temperature, and nuclei were stained with 4'-6-diamidino-2-phenylindole (DAPI) (Sigma, D9542, Santa Clara, CA, USA). Images were acquired on an Olympus F1000 confocal microscope.

Hematoxylin and eosin staining

The slices were treated with a hematoxylin and eosin staining kit (Beyotime, C0105, Wuhan, China). The slices were stained with hematoxylin for 8 min for P0 tissues or 20 min for P56 tissues, and all tissues were stained with eosin for 5 s. The thickness of the neo-cortex was measured using ImageJ software.

Quantitative real-time PCR assays

Total cortical RNA from P0 rats or the hippocampus of P56 rats was subjected to extraction using TRIzol (Invitrogen, 15596026, Carlsbad, CA, USA). Reverse transcription and first-strand cDNA synthesis were performed using the PrimeScript™ RT reagent Kit (TaKaRa, RR047A, Japan). Quantitative real-time PCR was carried out with the EvaGreen dye (Biotium, catalog # 31000, USA). The primers used in the qRT-PCR assays were specific for neurogenesis-associated genes. The primer sequences are listed in Supplemental Table 2.

Statistical analyses

Statistical calculations were conducted using GraphPad Prism 5 software. In the statistical graphs, the error bars represent the s.e.m. Statistical significance was determined using Student's T-test, the Mann-Whitney test or one-way ANOVA, and P values <0.05 were considered statistically significant.
